# The effectiveness of palliative middle meningeal artery embolization prior to craniotomy for large acute epidural hematoma: A case report

**DOI:** 10.1016/j.ijscr.2024.109704

**Published:** 2024-04-24

**Authors:** Yuichi Hirata, Yu Takahashi, Satoshi Kuramoto, Shingo Nishihiro, Yasuhiro Ono, Tomotsugu Ichikawa

**Affiliations:** a Department of Neurological Surgery, Okayama University Graduate School of Medicine, Dentistry and Pharmaceutical Sciences, Okayama, Japan; b Department of Neurological Surgery, Kagawa Prefectural Central Hospital, Takamatsu, Kagawa, Japan

**Keywords:** Acute epidural hematoma, Middle meningeal artery embolization, Transcatheter arterial embolization

## Abstract

**Introduction and importance:**

Acute epidural hematoma is typically managed with craniotomy. However, there are a few reports on transcatheter arterial embolization (TAE) as an adjunctive therapy.

**Case presentation:**

A 70-year-old female with no obvious history of trauma was transported to our hospital. Computed tomography scan revealed an epidural hematoma of approximately 80 ml with a midline shift of 5 mm. We decided to perform an emergency craniotomy. However, the operating room (OR) was already occupied by a scheduled surgery and it would take 30 min to an hour to prepare it. We opted to wait for our OR, considering that, even if the patient was transferred to another hospital, it would take time for the craniotomy to commence.

**Clinical discussion:**

We performed TAE for the middle meningeal artery (MMA) as a palliative measure to prevent hematoma enlargement. The MMA was selectively embolized with 20 % n-butyl-2-cyanoacrylate (NBCA), resulting in no hematoma enlargement or observed complications. The criteria for endovascular treatment of acute epidural hematoma are not yet well-established. This case demonstrates the potential role of endovascular treatment for large acute epidural hematomas in carefully selected patients.

**Conclusion:**

If there is a time gap before craniotomy, TAE could be considered a viable option for large acute epidural hematomas as a palliative intervention before craniotomy.

## Highlights

• Large acute epidural hematoma should be promptly treated with craniotomy.
• However, there are situations where immediate craniotomy is not possible because the operating room is not available.
• In cases where craniotomy is impossible, palliative transcatheter arterial embolization embolization (TAE) could be a palliative treatment option.

## 1 Introduction

Acute epidural hematoma is a life-threatening emergency condition. In this case report, we present a case of acute epidural hematoma in which we performed a palliative middle meningeal artery embolization to prevent hematoma enlargement before proceeding to craniotomy for hematoma removal. The case presented herewas managed at a tertiary academic center and was reported in line with SCARE criteria [1].

## 2 Presentation of case

This is the case of a 70-year-old woman with no significant medical history. Initially found sitting on the sidewalk next to her bicycle, she complained of a headache when approached by a passerby who called for an ambulance. Upon arrival at our hospital, her neurological examination showed no focal deficit and her Glasgow Coma Scale score was 12 (E3V4M5). Pupils were measured at 3.0 mm with a prompt light reflex on both sides. While she was initially able to say her name and discuss her headache, her consciousness gradually declined, and incomplete paralysis on her right side became evident.

**Fig. 1:**
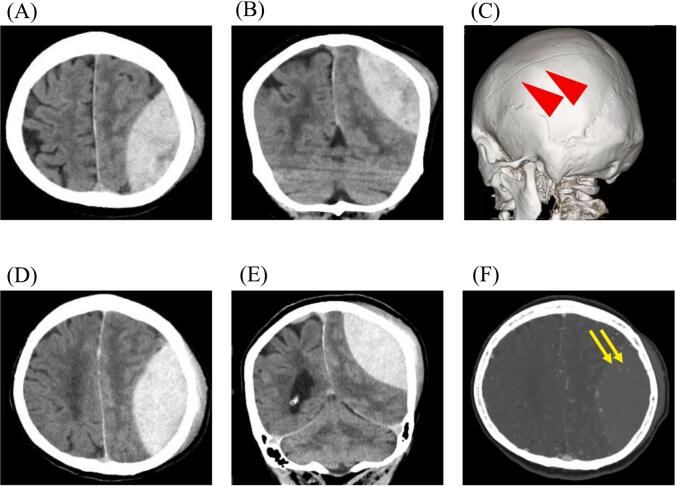
Initial brain computed tomography (CT) at admission. Noncontrasted axial (A) and coronal (B) CT showed an acute epidural hematoma with a thickness of 40 mm and a volume of approximately 80 ml. An approximately 5 mm midline shift was also observed. The three-dimensional image (C) showed a temporal bone fracture. Fifty minutes after the initial CT scan, axial (D) and coronal (E) images indicated a tendency for hematoma enlargement. Contrast-enhanced CT (F) showed numerous spot signs in the hematoma cavity, suggesting persistent bleeding.

A brain computed tomography (CT) scan revealed an epidural hematoma with a thickness of 40 mm and a volume of approximately 80 ml. A midline shift of approximately 5 mm was observed, along with a temporal bone fracture and subarachnoid hemorrhage. At this point, there was no apparent brain contusion, and no obvious traumatic changes observed in her trunk. Follow-up CT scans revealed the enlargement of the hematoma, and a contrast-enhanced CT revealed a spot sign, indicative of persistent bleeding (Fig. 1). Blood tests indicated that D-dimer level was 223 μg/ml and fibrinogen was 180 mg/dl which suggested coagulopathy.

We considered emergency craniotomy, but the routine operating room (OR) was not available because of a scheduled surgery and our hospital didn't have an emergency OR. We considered transferring her to another hospital for craniotomy, but our OR could be prepared within 30 min to an hour because there was another surgery that seemed to be finishing soon. Given the potential delay if transferred, we opted to wait for our OR to minimize the time until the start of craniotomy. Although we contemplated making a burr hole in the emergency room for evacuation of the hematoma, concerns about uncontrollable hemorrhage due to the spot sign and coagulopathy made us hesitate.

With time available before craniotomy, we decided to perform transcatheter arterial embolization (TAE) for the middle meningeal artery (MMA) in the angiography room as a temporary measure. The plan was to start craniotomy as soon as the OR was ready.

**Fig. 2:**
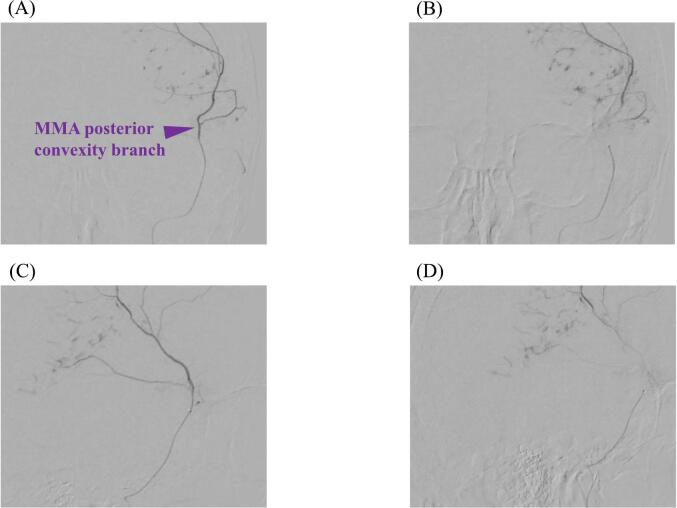
Super selective contrast images of the posterior convexity branch of the middle meningeal artery (MMA). Anteroposterior view (A and B) and lateral view (C and D) revealed extravasation of contrast media.

**Fig. 3:**
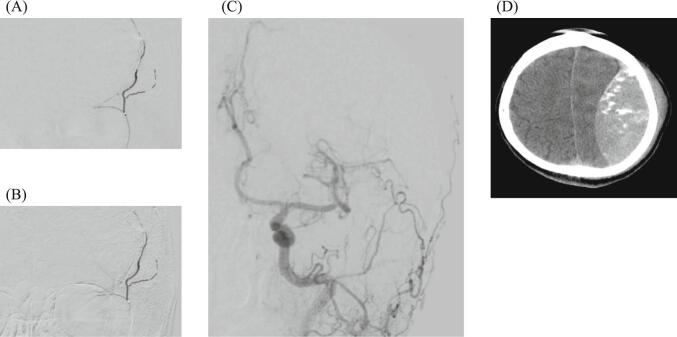
Embolization by NBCA. Injection of 20 % NBCA and embolization of the posterior convexity branch of the MMA (A and B). Angiography after TAE (C) showed the disappearance of extravasation of contrast media. CT in the angiography room (D) showed contrast leakage in the blood cavity, but no significant hematoma enlargement.

Six minutes after entering the angiography room, a 5Fr long sheath was placed in the right femoral artery. Heparin was not used due to bleeding concerns. A 5 Fr ENVOY (Johnson & Johnson, Miami, FL, USA) was placed at the left external carotid artery. Angiography showed contrast media extravasation from the posterior convexity branch of the MMA, prompting the decision to embolize the artery (Fig. 2). A micro-catheter (Renegade; Boston Scientific Corporation, Boston, MA, USA) was positioned immediately before the extravasation site using a 0.014-in. micro-guidewire (Asahi Chikai 014; Asahi Intecc Co. Ltd., Aichi, Japan). We performed TAE with 20 % n-butyl-2-cyanoacrylate (NBCA) 23 min after groin puncture (Fig. 3). Subsequent angiography showed the disappearance of contrast media extravasation, and CT revealed no hematoma enlargement. As the OR became available toward the end of TAE, the sheath was not removed, and the patient was promptly transferred to the OR. The entire procedure, from entering the angiography room to leaving, took 40 min.

A linear skin incision was made from the superior edge of the zygomatic bone to the parietal region. First, a burr hole was created, and the hematoma was aspirated as much as possible, revealing no hematoma eruption. Subsequently, a circular small craniotomy was performed using a bone cutter, intersecting the fracture line. The hematoma was removed and the bleeding from the MMA had stopped. A small incision was made in the dura mater to confirm the absence of hematoma in the subdural space. The dura mater was lifted up to the bone flap to prevent epidural hematoma. A subcutaneous drain was placed, and the operation concluded with an operative time of 65 min and a bleeding volume of 50 ml.

**Fig. 4:**
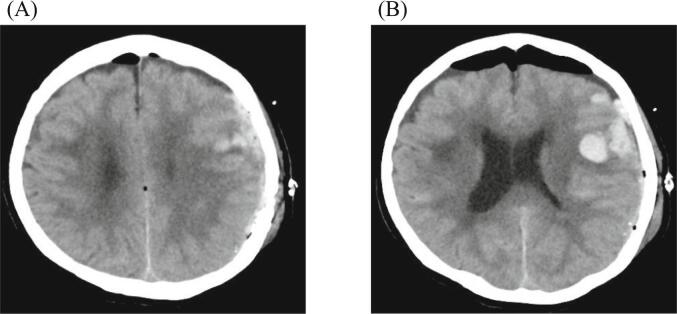
Follow-up CT on day 1 (A and B). Hematoma decompression was confirmed after surgery, and a brain contusion was evident from the left frontal lobe to the temporal lobe. Subsequent images showed no increase in the hematoma. Magnetic resonance imaging revealed an old brain contusion.

The following day, CT revealed no hematoma enlargement, confirming successful decompression. Cerebral contusions in the left frontal and temporal lobes were also observed (Fig. 4). As her consciousness gradually improved, the tracheal tube was removed. The patient began to speak and follow instructions. Rehabilitation was continued at our hospital and she was transferred to a rehabilitation hospital on the 19th day post-surgery with a Glasgow Outcome Scale (GOS) indicating moderate disability. After 3 months of rehabilitation, she was discharged home and continued rehabilitation as an outpatient. Five months after the injury, she maintained consciousness with mild motor aphasia and higher brain dysfunction, achieving a GOS score indicating good recovery, and resumed her normal life at home.

## 3 Discussion

Acute epidural hematoma is a critical neurosurgical emergency, accounting for 14 to 35 % of severe brain injuries [[2], [3], [4]]. Most cases are unilateral and 75 % are associated with a linear skull fracture [5]. The source of bleeding can involve the MMA, diploic vein, or venous sinus. Acute epidural hematoma is one of the most common intracranial traumatic lesions to require emergent surgery, usually under general anesthesia. In general, cases with a hematoma volume of 30 ml or more, a hematoma thickness of 15 mm or more, and a midline shift of 5 mm or more are indicated for craniotomy [6]. Rosario et al. indicated that conservative therapy may be considered in cases with a hematoma volume of 30 ml or less, a hematoma thickness of 25 mm or less, and a midline shift of 12 mm or less [7].

Coagulopathy is commonly observed after trauma and is associated with brain injury severity. A meta-analysis identified higher D-dimer levels at admission as a significant risk factor for hematoma enlargement [8]. Cases with high D-dimer or low fibrinogen tend to be more severe and have a higher incidence of hematoma enlargement [9]. Basamh et al. reported that the percentage of cases that initially received conservative therapy for acute epidural hematoma but subsequently required craniotomy was 11.2 % [10].

Endovascular treatment, commonly used for various conditions, has been applied in trauma patients for issues like traumatic carotid cavernous fistulas, pseudoaneurysms, and intractable bleeding. Reports exist of endovascular embolization for small acute epidural hematomas, demonstrating no postoperative complications [[11], [12], [13], [14]]. In a previously reported case, we performed MMA embolization for a small acute epidural hematoma in a 65-year-old man who had sustained an injury from a fall. CT revealed a thin epidural hematoma localized in the right middle cranial fossa at admission that showed a slight increase 3 h after admission. Due to the potential for further enlargement and the unavailability of the OR at the time, we decided to perform TAE. The MMA was selectively embolized with 18 % NBCA, and no hematoma enlargement was observed. No postoperative complications arose, and craniotomy was avoided in this case.

Endovascular treatment for acute epidural hematoma was reported by Suzuki et al. in 2004, where they successfully treated nine cases [15]. Their reported cases had hematoma thicknesses ranging from 8.5 mm to 20 mm. In our cases, the hematoma thickness was 40 mm, which was twice as thick as theirs.

Peres et al. reported on 80 cases in which MMA embolization was performed for small acute epidural hematomas in 2018 [16]. Cases with midline shift or skull fractures were excluded. No hematoma enlargement or postoperative complications were observed after TAE in any of the 80 cases. They reported the efficacy and safety of MMA embolization in preventing hematoma enlargement. In our case, angiography showed contrast media extravasation from the posterior convexity branch of the MMA, prompting the decision to embolize the artery. Further bleeding was critical for the patient, so if there had not significant contrast media extravasation, we would have embolized MMA because embolizing the MMA carries a low risk of complications and bleeding originates from the MMA in most cases. The entire procedure took 40 min in our case. This may be difficult only in facilities in which endovascular surgery, especially thrombectomy is routinely performed.

Ohshima et al. reported a case of acute epidural hematoma treated with a combined approach involving endovascular embolization and endoscopic surgery under local anesthesia [11]. There was no bleeding after MMA embolization, and postoperative decompression and consciousness were achieved. They reported the effectiveness of MMA embolization and burr hole surgery for hematoma removal under local anesthesia as less invasive treatments. Kim et al. reported a successful case in which they performed MMA embolization because of difficulty in hemostasis during craniotomy. After performing angiography, they embolized MMA with NBCA and were able to stop the bleeding [17].

Based on previous experiences and reports on preventing hematoma enlargement and avoiding surgery by performing MMA embolization for small acute epidural hematomas, we performed MMA embolization in this case as a palliative treatment. The goal was to prevent hematoma enlargement and to buy time until craniotomy. Successful TAE helped prevent cerebral herniation.

Use of anticoagulants during TAE for acute epidural hematomas are not mentioned in the previous reports. Hundersmarck et al. reported 135 cases of blunt pelvic trauma treated with TAE. Heparin was used in only 25 cases due to the risk of heparin administration, and TAE related ischemic complications were observed in only 4 cases [18].

There is a report suggesting that burr hole surgery is effective for slowly developing acute epidural hematomas [19]. Patients with coagulopathy have a higher risk of intraoperative bleeding, making less invasive burr hole evacuation advantageous due to fewer intraoperative hemorrhagic complications. For patients with coagulopathy, burr hole evacuation and drainage may be a more feasible and safer treatment option. However, in our case, the hematoma volume was larger than that of previous reports, and the rapid expansion of the hematoma made hematoma removal and burr hole surgery under local anesthesia challenging. Thus, we opted for a large craniotomy under general anesthesia. To the best of our knowledge, this is the first report of MMA embolization for a large acute epidural hematoma. No complications were observed after MMA embolization, and we were able to start craniotomy without any problems.

The criteria for endovascular treatment of acute epidural hematoma are not yet well-established. If the patient has an imminent hernia, it might be necessary to buy time by decompression by perforation before TAE. This case demonstrates the potential role of endovascular treatment for large acute epidural hematomas in carefully selected patients. If there is time until craniotomy, in situations where patients don't have imminent hernia and the imaging findings suggest the persistent hemorrhage, we believe that TAE is a viable palliative treatment option for large acute epidural hematomas before craniotomy while craniotomy is the most important treatment and any surgical delays should be avoided.

## 4 Conclusion

We experienced a case of large acute epidural hematoma, where we successfully prevented hematoma enlargement through palliative MMA embolization before craniotomy. The optimal size for safely evacuating a large hematoma via a small craniotomy or burr hole remains uncertain. In our experience, following MMA embolization, the large acute epidural hematoma showed no active hemorrhage during the initial burr hole procedure. In situations where there is time until craniotomy, in situations where patients don't have imminent hernia and the imaging findings suggest the persistent hemorrhage, we believe that TAE is a viable treatment option for large acute epidural hematomas as a palliative intervention before craniotomy.

## Consent

Written informed consent was obtained from the patient for publication of this case report and accompanying images. A copy of the written consent is available for review by the Editor-in-Chief of this journal on request.

## Provenance and peer review

Not commissioned, externally peer-reviewed.

## Ethical approval

Ethical approval was not required for this case report.

## Funding

This research did not receive any specific grant from funding agencies in the public, commercial, or not-for-profit sectors.

## Author contribution

All authors contributed in writing the paper.

## Guarantor

Yuichi Hirata.

## Research registration number

Not applicable.

## Conflict of interest statement

None.
